# Upscale fermenter design for lactic acid production from cheese whey permeate focusing on impeller selection and energy optimization

**DOI:** 10.1007/s13197-021-05239-6

**Published:** 2021-08-25

**Authors:** Maneesh Kumar Mediboyina, Nicholas M. Holden, Simon O’Neill, Kai Routledge, Bill Morrissey, Fergal Lawless, Fionnuala Murphy

**Affiliations:** 1 UCD School of Biosystems and Food Engineering, University College Dublin, Belfield, Dublin, Ireland; 2 School of Biomolecular and Biomedical Science, University College Dublin, Belfield, Dublin, Ireland; 3 BiOrbic Bioeconomy SFI Research Centre, University College Dublin, Belfield, Dublin, Ireland; 4 Glanbia Ireland DAC, Lisheen Mine, Moyne, Dublin, Thurles, Co. Tipperary Ireland

**Keywords:** Dairy cheese whey, Lactic acid, Fermenter scale-up, Power consumption

## Abstract

This study focusses on the design and scale-up of industrial lactic acid production by fermentation of dairy cheese whey permeate based on standard methodological parameters. The aim was to address the shortcomings of standard scale-up methodologies and provide a framework for fermenter scale-up that enables the accurate estimation of energy consumption by suitable selection of turbine and speed for industrial deployment. Moreover, life cycle assessment (LCA) was carried out to identify the potential impacts and possibilities to reduce the operation associated emissions at an early stage. The findings showed that a 3000 times scale-up strategy assuming constant geometric dimensions and specific energy consumption (*P/V*_w_*)* resulted in lower impeller speed and energy demand. The Rushton turbine blade (RTB) and LightninA315 four-blade hydrofoil (LA315) were found to have the highest and lowest torque output, respectively, at a similar *P/V*_w_ of 2.8 kWm^−3^, with agitation speeds of 1.33 and 2.5 s^−1^, respectively. RTB demonstrating lower shear damage towards cells (up to 1.33 s^−1^) was selected because it permits high torque, low-power and acceptable turbulence. The LCA results showed a strong relation between the number of impellers installed and associated emissions suggesting a trade-off between mixing performance and environmental impacts.

**Supplementary Information:**

The online version contains supplementary material available at 10.1007/s13197-021-05239-6.

## Introduction

Whey is a major by-product of cheese and casein manufacturing with a global production of 180 million tonnes per annum. Approximately 40% of global whey is disposed of as dairy effluent (Panghal et al. 2018) due to its high chemical oxygen demand (COD) (50–80 g L^−1^) and biological oxygen demand (BOD) (40–60 g L^−1^) that can lead to adverse environmental impacts (Torres et al. 2019). Cheese whey can be valorised to recover high value biochemicals such as proteins through advanced filtration techniques (Deshwal et al. 2021; Argenta et al. 2021). However, the leftover permeate still contain the same lactose concentration as cheese whey. Therefore, it requires effective treatment before discharge into the environment. Fermentation process offers a solution for reducing the organic load to mitigate polluting potential whilst delivering high value biochemicals (Beitel et al. 2020).

Lactic acid (LA) is widely known for its application in food, pharmaceutical, medical and manufacturing industries, resulting in a tenfold increase in worldwide demand from 2010 to 2020 (Nolasco-Hipolito et al. 2019). The LA monomer can be used for the production of biodegradable polymers such as polylactic acid (PLA), which is an alternative for fossil-based polymers (Beitel et al. 2020). The market value of fermentation derived bioproducts is expected to increase to €5.2 billion by 2030 with a sustained demand for packaging. Such consumption estimates forecast the establishment of 45 new bioplastic production plants by 2030 with a capacity of 50 kt per year to meet the demand (BIO‐TIC 2015). One of the major reasons for projected growth is the success of viable pilot scale fermentation studies and technologies for LA production from different sources such as soyabean vinasse (Karp et al. 2011), sugar molasses and corn steep liquor (Beitel et al. 2020), sago starch (Nolasco-Hipolito et al. 2019), oil palm fruit bunch (Hassan et al. 2017) along with reliable scale-up methodologies for fermentation processes (Junker 2004).

Scaling up fermentation is usually carried out using theory, models and the principle of similarity (Meyer et al. 2017). The scale-up methods are based on considering constant fermenter characteristics such as geometry, impeller tip speed, mixing time and specific energy consumption as key criteria (Junker 2004). Though all the scale-up characteristics are important, it has been suggested that for specific scale-up by keeping a particular set of parameters constant, the other parameters may vary considerably (Junker 2004). For instance, during fermenter scale-up involving viscous liquid systems, the specific energy consumption (*P/V*_*w*_) can increase by (*V*_*t*_)^2/3^ to maintain equal mixing times (Meyer et al. 2017), where *V*_*t*_ is the total volume of fermenter. This increase leads to an overestimation of power consumption of the large-scale system compared to that expected in practice (Junker 2004). Therefore, the bioprocess system must be assessed to identify the range of fermenter characteristics that can be kept constant during scale-up and place them in a sequence that is critical for bioprocess performance (Ju and Chase 1992).

The fermentation process is regarded as one of the most energy intensive processes during the production of biobased products from whey (Koller et al. 2013). The scaling up from a pilot-scale fermenter is helpful in early identification and understanding of process bottlenecks and environmental threats (Piccinno et al. 2016). Companies like Coskata, INEOS Bio, and LanzaTech have implemented pilot and demonstration gas fermentation facilities in order to optimize the technology (focussing on bioreactor design and its performance) for conversion of syngas to ethanol before advancing to commercial scale plants (Heijstra et al. 2017). The environmental analysis is generally performed using life cycle assessment (LCA), which can be used to influence decision-making in the early stages of process design (Piccinno et al. 2016).

Industrial scale-up studies based on real-time operational information are scarce for dairy whey permeate fermentation processes. Moreover, available scale-up studies for LA production are mostly linked to aerobic fermentation that employs oxygen transmission rate (OTR) or k_L_a as the strategy and employs standard scale-up methodologies (Hassan et al. 2017; Junker 2004; Meyer et al. 2017; Ju and Chase 1992) The research presented is for the design and scale-up to 300 m^3^ of a continuous stirred-tank reactor (CSTR) anaerobic fermenter for LA production from dairy cheese whey permeate from 0.03 m^3^ laboratory and 0.1 m^3^ pilot scale processes data. The strategy used for scale-up assumes similar geometric dimensions across the different scales for fermenter design and considers onsite industrial information for the impeller design. However, this study also emphasises the discrepancies between conventional scale-up results and industrial norms, consequently recommending a particular design for the problem at hand to estimate the fermenter energy consumption by selecting suitable impeller type and speeds. The impeller type and speed were estimated based on criteria of high torque output, low mixing energy and acceptable turbulence among commercial turbine designs operating within design limits for impeller speed. The scale-up is part of a first-of-a-kind industrial scale biorefinery to use cheese whey permeate as the feedstock to produce bio-based lactic acid (LA).

## Materials and methods

### Laboratory and pilot scale fermentation

The fermentation seed was prepared by inoculating a glycerol stock containing a *Bacillus sp.* inoculum, into a 100 mL enriched media flask left overnight and shaken in an incubator at 3.33 s^−1^ under optimised conditions. The 100 mL flask was inoculated into 500 mL flasks with enriched media under the same conditions and left until the cells were in a logarithmic growth phase, at which point the cells were transferred into the pilot-scale starter culture tanks. LA production fermentations were carried out with the substrate and seed in both 0.03 m^3^ laboratory and 0.1 m^3^ pilot scale fermenters (Fuente Alamo, Bionet, Spain). Mixed dairy cheese whey permeate (Glanbia Ireland, Ireland) was used as the LA fermentation substrate with a total solids content of 16% (ww^−1^). A 4% seed inoculum (vv^−1^) was used to inoculate the laboratory scale fermenter from the inoculum flasks and performed under optimal conditions at 48 °C, agitation set at 3.33 s^−1^ and controlled at pH 6.5 (maintained by an automatic feed using 23% (ww^−1^) Ca(OH)_2_ as the titrant). Subsequently, the same conditions were used for the pilot scale LA production fermentation. The culture medium for the LA production fermentations contained 5 g L^−1^ yeast extract based on the final fermentation volume. The fermentation process was developed and patented by Glanbia Ingredients Ireland for lactic acid production from whey permeate in an industrial perspective (O'connor et al. 2021). Therefore, technical details like optimised cell conditions, logarithmic log phase and cell concentrations etc., are deemed to be commercially sensitive and is not provided in the paper on the request of project partners and stakeholders.

### Analytical methods

The viscosity, density and total solids of each dairy whey sample was measured in triplicate to determine the average result. The dynamic viscosity of the samples was measured with a VISCO-895 viscometer (ATAGO, Japan). The viscosity was measured at 4.16 s^−1^ at room temperature. The density was found using a BP 211D electronic analytical balance (Sartorious AG, Germany) using 1 mL sample as the reference volume for each test. The total solids (%) of the samples were measured using a LECO TGA 701 thermogravimetric analysis instrument (TGA) (LECO Corporation, USA). The TGA start and end temperature was set at 25 °C and 107 °C, respectively (Eq. 1):

1 $$ Total\,solids\,\left( \% \right) = \frac{Initial \,mass\, \left( g \right) - Moisture\, mass\, \left( g \right)}{{Initial \,Mass\, \left( g \right)}} x 100\% $$

### Design of the fermenter

The outcomes associated with pilot and lab scale studies were then extrapolated to a large-scale industrial CSTR fermenter. The AgriChemWhey project (Grant number 744310) proposed to build a 20,000 t yr^−1^ capacity LA production plant for valorising dairy side streams. At this stage, only pilot plant testing data were available. The fermentation process was assumed to run for 20 h per day and 7 days a week delivering 400 tonnes of LA week^−1^, which corresponds to 57 t day^−1^. A fermentation lactose to LA yield of 95% was assumed for the industrial scale plant operational for 320 d yr^−1^. The industrial CSTR fermenter required a holding capacity of 300 m^3^ with specifications provided by experts associated with the AgriChemWhey biorefinery project. The CSTR fermenter design is presented in supplementary material (Fig. S1). The fermenter details (tank capacity (*V*_t_), diameter (*T*), working volume (*V*_w_), number of baffles and blades) were obtained from the laboratory and pilot plant setup. Dimensional characteristics of the large scale CSTR were based on the assumptions reported in Meyer et al. (2017) and Fitschen et al. (2019) and represented in detail in supplementary material (Table S1).

The motor installed for mixing is usually the most energy demanding component during the fermentation process (Meyer et al. 2017). The power consumption of a single impeller motor during the mixing operation can be calculated using (Pietranski 2012) (Eq. 2):

2 $$ P = \frac{{\left( {P_{o} *N^{3} * D^{5} * {\rho }} \right)}}{{G_{c} }} $$

where *P* (W) is mixing power, *P*_o_ is the power number of the impeller, *N* is impeller rotation speed (s^−1^), *D* is the impeller diameter (m), ${\rho }$ is the density (kg m^−3^) and *G*_c_ is the Newton-law proportionality factor (assumed to be 1 N s^−2^ kg^−1^ m^−1^). A two-stage Rushton turbine was assumed to calculate the mixing power across all the scales of fermenter. For calculating the power drawn by the motor the correlation between Power number (*P*_o_) and Reynolds number (*R*_e_) for agitated batch liquids (Pietranski 2012) was used. Reynolds number (*R*_*e*_) indicates the flow of momentum associated with the bulk motion of the liquid (Eq. 3):

3 $$ R_{e} = \frac{{\rho {\text{*N*D}}^{2} }}{\mu } $$

where $\mu$ is liquid viscosity (N·s m^−2^). The relationship between *P*_o_ and *R*_e_ for different types of impellers was taken from Hemrajani and Tatterson (2004) for laminar, transition and turbulent flow regimes. The characteristic *P*_o_ values of the impellers were ascertained after identifying the nature of mixing fluid regimes by using Eq. 3.

The number of impellers also has a significant impact on power consumption during mixing. The number of impellers was calculated using (Fitschen et al. 2019) (Eq. 4):

4 $$ \frac{{\left( {Z - D} \right)}}{D} > No.~\;of~\;impellers~\;\left( n \right) > \frac{{\left( {Z - 2D} \right)}}{{2D}} $$

where *Z* is fermenter working volume height (m). The number of impellers required for the industrial fermenter was estimated as two-stage and three-stage for different *Z* values (5.99 and 9.29 m) using Eq. 4. It is generally assumed that power dissipated by a multiple stage Rushton turbine with optimal space results in the sum of power dissipated by a single impeller (Petříček et al. 2018). Subsequently, the power required was doubled and tripled due to the usage of a two-stage and three-stage Rushton turbine system, respectively. Torque is an important design parameter as it determines the impeller mixing power consumption. The torque (*T*_*q*_, N⋅m) required was calculated using (Ylinen 2005) (Eq. 5):

5 $$ T = \frac{P \times 63025}{N} \times 0.112985 $$

Proper mixing of the fermentation medium is based on turbulence caused within the liquid which occurs due to the transfer of rotational energy around the impeller blade to kinetic energy. This implies that the energy is dissipated into the fluid at a rate equal to impeller motor power. So, for a stirred fermenter vessel of working volume (*V*_w_) the average power transmitted to the liquid per unit mass (*ε*_*t*_), also called the specific energy dissipation rate, as calculated as (Eq. 6):

6 $$ \varepsilon _{t}  = \frac{P}{\rho } \times V_{t}  $$

The Kolmogoroff microscale of turbulence (*λ*_*k*_) is dependent on energy dissipated in the fermenter and liquid kinematic viscosity (*µ*_k_), which can be indicative of mechanical damage to microbial cells (Stitt and Simmons 2011) and was calculated using (Eq. 7):

7 $$ \lambda _{k}  = ~\left( {\frac{{\varepsilon _{t} }}{{\left( {\mu k} \right)^{3} }}} \right)^{{ - 1/4}}  $$

### Scale-up criteria

The scale-up was required to estimate the characteristics of an industrial-scale CSTR fermenter based on the optimised pilot or laboratory scale process. It was accomplished by reviewing scale-up strategies suggested by various authors (Hassan et al. 2017; Junker 2004; Meyer et al. 2017; Ju and Chase 1992; Piccinno et al. 2016; Heijstra et al. 2017) and selecting those most appropriate for the task, which depended on vessel type, process type (aerobic or anaerobic, gassed or ungassed) and existence of primary data. It is generally accepted to have a combination of constant parameters for scale-up design processes in bioreactors as the inter-dependency of these characteristics affects the other parameters in unanticipated ways (Ju and Chase 1992). The research assumed geometric similarity and constant specific energy consumption (*P/V*_w_) as a characteristic combination for scale up across the different scales of fermenter. Initially, the most important criterion was to achieve geometric similarity for all fermenter sizes (Junker 2004) represented as (Eq. 8):

8 $$ \frac{{T_{2} }}{{T_{1} }} = \left( {\frac{{Z_{2} }}{{Z_{1} }}} \right)^{1/3} $$

Geometric similarity of fermenters at different scales (0.003, 0.1, 300 m^3^) presumes similar impeller diameter and number of impellers (n) along with various dimensionless parameters such as total slurry height ratio (*Z*/*T*), total tank height ratio (*H/T*) and impeller diameter ratio (*D/T*). The geometric dimensional comparison for the different scales of fermenters is shown in Table 1.

**Table 1:** Geometric and scale-up comparisons for different scales of fermenter

Fermenter working volume (*V*_w,_ m^3^)	Impeller diameter ratio (*D/T*)	Total height ratio (*H/T*)	Geometric similarity at *T*/(*Z*)^1/3	Actual working volume height (*Z*_0_, m)	Actual liquid height ratio (*Z*/*T*)	Geometric similarity at *Z*	Calculated working volume height (*Z*)	Calculated (*Z*/*T*) used
0.003	0.45	3	1 at 0.003 m^3^	0.18	1.37	1	0.18	1.37
0.1	0.45	3	0.81 at 0.1 m^3^	0.65	1.61	1	0.53	1.32
207	0.45	3	0.53 at 207 m^3^	11.47	2.55	1	5.99 (9.29*)	1.33

*Z* is the fermenter working volume height

*value based on pilot scale (0.1 m^3^) with geometric similarity ratio (0.81)

In the next stage of scale-up, the turbulent mixing behaviour (*E*_t_) was assumed to be constant across the fermenter liquid volume. This scaling principle focuses on keeping similar mixing performances at all scales, which is essential for complex biological reactions where selectivity of the desired product is vital. For scaling up an ungassed stirred tank reactor, power required per unit volume of liquid (*P/V*_w_) can be represented as (assuming constant fluid density ($\rho$)) (Eq. 9)

9 $$ \frac{{\left( {\frac{P}{{V_{w} }}} \right)_{2} }}{{\left( {\frac{P}{{V_{w} }}} \right)_{1} }} = \left( {\frac{{V_{w1} }}{{V_{w2} }}} \right)^{0.37} $$

The value 0.37 was based on a study conducted by Ju and Chase (1992), involving industrial plants at different scales and process complexity. Usually, the power number (*P*_o_) is constant when the flow is turbulent (*R*_*e*_ > 10,000). The power required during agitation is given by Eq. 3. During bioreactor scale-up with similar tank design, and impeller design (Junker 2004), the equation is reorganized as (Eq. 10):

10 $$ \frac{{N_{2} }}{{N_{1} }} = \left( {\frac{{V_{w2} }}{{V_{w1} }}} \right)^{0.21} x \left( {\frac{{D_{1} }}{{D_{2} }}} \right)^{5/3} $$

The turbulent nature of fluid (*R*_*e*_ > 10^4^) in both 300 m^3^ (2.5 × 10^6^) and 0.1 m^3^ (2.04 × 10^4^) fermenters allowed for the calculation of impeller speed (*N*) for the 300 m^3^ fermenter using Eq. 10. The *P*_o_ value (5) for turbulent flow was determined from *R*_*e*_ vs *P*_o_ power correlation graph (Stitt and Simmons 2011). Consequently, the mixing power consumption (P) for 300 m^3^ fermentation was calculated using Eq. 2. The scaled-up *N* and *P* values for 300 m^3^ fermenter were compared with the pilot scale fermenter and literature values to examine its practical suitability. Moreover, six different impeller types were selected combining five different agitation speed to design a suitable impeller type and speed for an industrial scale fermenter. The selected impeller types (Rushton turbine blade (RTB), Lightnin A310 three blade hydrofoil (LA310), Lightnin A315 four-blade hydrofoil (LA315), concave blade turbine (CBT), pitched blade turbine (PBT) and Marine propeller (MP)) are widely employed for mixing the fermentation liquids (Markopoulos and Pantuflas 2001; Stitt and Simmons 2011; Petříček et al. 2018). The selected agitation speeds (0.83, 1.33. 1.83 and 2.5 s^−1^) were based on the reported values in the studies (Hassan et al. 2017; Meyer et al. 2017; Ju and Chase 1992) that focussed on lactic acid production in stirred tank fermenters. The agitation speed was the estimated value for the 300 m^3^ fermenter using Eq. 10, where total tank volume (*V*_*t*_) and impeller diameter (D) for both 0.1 m^3^ and 300 m^3^ fermenters are known along with agitation speed (*N*_*1*_) of the 0.1 m^3^ fermenter (Table 1). Meyer et al. (2017) suggested general values (2–4 kWm^−3^) for specific energy consumption (*P/V*_w_) for industrial scaled up fermenter operations. This range was used as rule of thumb to estimate the motor size for this research.

### Life cycle assessment (LCA)

The LCA methodology was performed in compliance with ISO 14044:2006, focused on the following stages: goal and scope, life cycle inventory and life cycle impact assessment (Finkbeiner 2014). Initially, the goal of the study was defined along with a set boundary for the system to be analysed. The inventory data of the system was collected and collated for inputs (material and energy) and outputs (emissions). Finally, the system associated emissions were transformed into respective impact categories by using emissions factors dictated with chosen impact assessment methodologies. The obtained LCA results were analysed and correlate the metrics with the fixed objectives (Finkbeiner 2014).

The goal considered the reason for the LCA, which was to understand the environmental impacts likely to be associated with the scaled-up industrial fermenter (300 m^3^ capacity). The application was to select from 4 turbine options (RTB, CBT, PBT and LA315) at different speeds (1.33, 1.83 and 2.5 s^−1^) to identify the lowest impact option. The audience were plant design engineers and management and there was no intention for public disclosure of specific product comparisons. Since the fermentation process is a major contributor to environmental impact within the production chain of whey-based products (Koller et al. 2013) and plays a potential role in determining the environmental performance of whole system, the scope was limited to the immediate design elements around this process. The choice of impeller design is independent of upstream and downstream components of the system, so a gate-to-gate system boundary was used. The system boundary encompassed the fermenter operation, and the functional unit was 1 m^3^ of substrate (dairy whey permeate) processed. The life cycle impact assessment was performed using CML (Institute of Environmental Sciences of Leiden University, 2001) methodology (Koller et al. 2013). The environmental impacts examined were acidification (AP), eutrophication (EP), freshwater aquatic ecotoxicity (FAETP), global warming (GWP 100 years), human toxicity (HTP), ozone layer depletion (ODP), photochemical ozone creation (POCP) and terrestrial ecotoxicity (TETP). The life cycle analysis was carried out using OpenLCA version 1.10.3.

The life cycle inventory was adopted from the optimised scale-up results (discussed in results and discussion section). The background data for electricity production were obtained from the Sustainable Energy Authority of Ireland (SEAI 2020) and Ecoinvent database (v3.1) considering geographic location as Ireland. In standard large scale fermenter operations, the working volume remains around 70–80% of the total fermenter volume (Stitt and Simmons 2011), which dictates the number of impellers to be used and subsequently the energy consumed. In other words, the reduction in *Z* values could reduce the number of impellers employed (Eq. 4) along with the fermenter energy demand. The working volume height (*Z*) and *H/T* ratio plays a major role in energy optimization as these two factors may affect the energy consumed by the turbine with different fermenter geometries. In this regard, a scenario analysis was used to understand the effect of *Z* values and *H/T* ratios on specific energy and global warming (GWP) of the 300 m^3^ fermenter using the optimized results (turbine type and speed) of the study.

## Result and discussion

The fermenter liquid density and viscosity across all the scales (laboratory, pilot and industrial) are presented in Table 2. The *D/T* and *H/T* ratios calculated based on the available market size fermenter dimensions were similar, whereas *Z/T* ratio differed across the different scales. From Table 1. it is evident that the calculated geometric similarity parameter at *Z* decreased with an increase in fermenter volume. To achieve the geometric similarity for larger scale (0.1 and 300 m^3^) to that of smaller scale (0.003 m^3^) process, the working volume height (*Z*) for 0.1 and 300 m^3^ capacity fermenters had been decreased from 0.65 and 11.47 m to 0.53 and 6 m, respectively. The results were in agreement with Junker (2004), where the author observed a decrease in working volume of a large-scale fermenter (19 m^3^ capacity) in order to attain a geometric similarity close to smaller scale vessels (0.1 and 0.28 m^3^). However, the working volume height (*Z*) for the 300 m^3^ fermenter (6 m) indicated 52% occupancy of the actual total height (11.47 m), which is below the standard acceptable working volume (80%) for fermenters (Stitt and Simmons 2011). Therefore, the geometric similarity ratio of the pilot scale fermenter (0.81) was applied to estimate the *Z* value for 300 m^3^ fermenter as 9.29 m to maintain the similarity coefficient between pilot and industrial scales. Usually, the change in *Z* values leads to deviation in the geometric similarity ratios. Consequently, effecting the number of impellers and power consumption. Meyer et al. (2017) suggests considering the scalable parameters at closest developmental stage of the process while scaling up bioprocesses to predict its behaviour at larger scales. Therefore, the power consumption for the industrial scale fermenter at working volume height (*Z*) at 9.29 m was also estimated and presented in Table. 3.

**Table 2:** Physico-chemical and hydrodynamic characteristics of fermenter liquid at difference levels

Parameters	Units	Lab scale	Pilot scale	Industrial scale
Fluid density (*ρ*)	kg m^−3^	1060	1060	1060
Dynamic viscosity of fluid (*µ*_d_)	kg m^−1^ s^−1^	0.0068	0.0057	0.0057
Kinematic viscosity of fluid (*µ*_k_)	m^2^ s^−1^	6.36 × 10^−6^	5.37 × 10^−6^	5.37 × 10^−6^
Specific energy dissipation rate (*e*_t_)	W kg^−1^	0.080	0.726	0.042
Kolmogoroff or microscale of turbulence, (*λ*_*k*_)	µ m	237.721	120.874	245.125 (221.495*)

*value based on vessel working volume height (Z) at 9.29 m

**Table 3:** Power consumption and hydrodynamic parameters of scaled up fermenters

Fermenter scale (m^3^)	Impeller speed^a^ (*N*, s^−1^)	Power input (*P*_*w*_, Watt)	Impeller speed (*N*, s^−1^)	Reynolds number (*R*_*e*_) × 10^4^	Power input (*P*_*w*_, Watt)	*P/V*_w_, Watt m^−3^
0.003	3.333^*b*^	0.279	3.333^*b*^	0.181	0.257	85.709
0.1	1.069^*c*^	2.545	3.333^*b*^	2.040	77.006	770.069
207	0.014^*c*^	1.134	0.295^*c*^	255.319	9425.137 (14,137.705*)	45.532 (68.29*)

*a*−estimated based on constant *P/V*w, *b*−based on AgriChemWhey onsite operational data; *c*−calculated based on Eq. 10; *values based on vessel working volume height (*Z*) at 9.29 m

The impeller speeds (*N*) were estimated based on Eq. 10 and presented in Table 3. Still, the *N* values were very low in comparison with both the 0.003 and 0.1 m^3^ fermenters operated at 3.33 s^−1^ at the pilot plant site. Therefore, a nominal agitation speed of 3.33 s^−1^ was studied for both laboratory and pilot scale (Table 3) as it has been widely used for lactic acid fermentation of whey at laboratory (Yun et al. 2003) and pilot scale (Park et al. 2002). The impeller speed for the industrial scale fermenter was estimated to be 0.29 s^−1^, which is around 10 times less than the installed lab/pilot scale fermenters speed (3.33 s^−1^) (Table 3). The reduction of agitation speed while scaling up fermenters is mainly practised to maintain sufficient shear stress between the impeller blades and vessel wall (Junker 2004). As a result, uniform mixing is achieved with minimum microbial cell damage (Nienow 2021). The industrial scale fermenter was found to have a lower specific energy or *P/V*_w_ value than lab and pilot scale fermenters (Table 3) and observed to be lower than the range of values (2–10 kWm^−3^) suggested by Meyer et al. (2017) for pilot scale fermentations. However, the variation can be attributed to the different assumptions investigated during the process of scale-up such as geometric similitude, fluid characteristics, type, size and number of impellers employed, motor power installed etc. (Junker 2004; Meyer et al. 2017; Koller et al. 2013).

The calculated agitation speed (*N*) and *P/V*_w_ values for industrial scale fermenter were 0.29 s^−1^ and 0.045 kWm^−3^, respectively. But the agitation speed was quite slow to provide sufficient homogenization and substrate utilization during LA fermentation when the stirrer speed is below 1.16 s^−1^ (Beitel et al. 2020). Reported agitation speeds for efficient LA production range from 0.83 to 2.5 s^−1^ (Hassan et al. 2017; Meyer et al. 2017; Ju and Chase 1992; Heijstra et al. 2017). The lower values of *N* and *P/V*_w_ values of an industrial scale fermenter may lead to underestimation of power consumption and installed motor capacity (Stitt and Simmons 2011). The Rushton turbine is considered the traditional impeller used in fermentation processes with a relatively high-power number (5) (Meyer et al. 2017), but its limitations drive towards its comparison with other impeller designs presented in Table 4. These impellers selected have lower *P*_o_ values and could certainly substitute the Rushton impeller operating at similar power profiles with certain processing advantages (Meyer et al. 2017). The desired selection of motor size for industrial scale fermenter depends on specific power requirement and torque output. Estimated torque (using Eq. 5) and *P/V*_w_ values for different impellers at different agitation speeds are shown in Table 4. The data demonstrate that impeller type and *P*_o_ values significantly impact the power dissipation in the fermenter fluid. The increase in *P*_o_ values of different impellers indicated a considerable increase in power consumption. Moreover, impeller agitation speed (*N*) also influences power consumption which is proportional to the product of impeller stirrer speed and impeller diameter, *N*^*3*^*D*^*5*^ (Stitt and Simmons 2011).

**Table 4:** Power requirement and torque generation in industrial scale fermenter employed with different types of turbines and impeller speeds

Impeller speed, s^−1^ (rpm)		Motor power (kW)					Toque (N-m)					*P/V*_w_ (kWm^−3^)				
		0.29 (17)	0.83 (50)	1.33 (80)	1.83 (110)	2.5 (150)	0.29 (17)	0.83 (50)	1.33 (80)	1.83 (110)	2.5 (150)	0.29 (17)	0.83 (50)	1.33 (80)	1.83 (110)	2.5 (150)
Turbine type	*P*_*o*_															
Ruston turbine blade	5	9.43	210.4	862.54 1293.81*	2242.72	5689.94	3532.38	29,976.89	76,775.40 115,163.1*	145,183.19	270,116.05	0.03	0.70	2.88 4.31*	7.48	18.97
Concave blade turbine	4.4	8.29	185.23	759.03	1973.60	5007.15	3108.50	26,379.67	67,562.35	127,761.20	237,702.12	0.03	0.62	2.53	6.58	16.69
Pitched blade turbine	1.64	3.09	69.04	282.91	735.61	1866.30	1158.62	9832.42	25,182.33	47,620.09	88,598.06	0.01	0.23	0.94	2.45	6.22
Lightnin A310 three blade hydrofoil	0.3	0.57	12.63	51.75	134.56	341.40	211.94	1798.61	4606.52	8710.99	16,206.96	0.00	0.04	0.17	0.45	1.14
Lightnin A315 four-blade hydrofoil	0.75	1.41	31.57	129.38	336.41	853.49 1280.23*	529.86	4496.53	11,516.31	21,777.48	40,517.41 113,955.2*	0.00	0.11	0.43	1.12	2.84 4.27*
Marine propeller: (1.0 pitch, *W/T* = 0.1)	0.34	0.64	14.31	58.65	152.51	386.92	240.20	2038.43	5220.73	9872.46	18,367.89	0.00	0.05	0.20	0.51	1.29

*values based on vessel working volume height (Z) at 9.29 m

The *P/V*_w_ values within the range of 2–4 kW m^−3^ (Meyer et al. 2017) were selected from Table 4 to identify the appropriate impeller type and speed for motor size. The RTB design would facilitate greater torque output than CBT and PBT. Usually, the greater torque was associated with greater energy consumption, which increased with an increase in agitation speed, but it is interesting to note that RTB was calculated to have 1.9 times greater torque output than LA315 at similar specific energy consumption (~2.8 or 4.31 kW m^−3^ at *Z* = 6 or 9.29 m) and lower agitation rate (1.33 s^−1^). Moreover, Nienow (2021) indicated that microbial cells may remain unaffected by the hydrodynamic stress if the cell size is smaller than Kolmogoroff microscale of turbulence (*λ*_k_), which was calculated using Eq. 6 and 7. The estimated *λ*_k_ value for 2.8 and 4.3 kW m^−3^ at 1.33 s^−1^ were 245.12 and 221.49 µm, respectively, for RTB, which is well above the microbial cell length range (0.9–1.9 µm) (Table 2), indicating the suitability of the selected tubine type and agitation speed with lower possibilities of shear damage to the cells in the fermenter. Therefore, RTB has been selected as the impeller type with an agitation speed of 1.33 s^−1^, which will facilitate efficient mixing with reduced hydrodynamic stress on microbial growth and optimal energy demand. The agitation speed of 1.33 s^−1^ for pilot scale fermentations was also employed by Karp et al. 2011 for LA production using soybean vinasse as the fermentation substrate. The installed motor capacity was determined by introducing a correction factor of 1.5, that accounted for efficiency losses in the motor, bearing and gearbox (Junker 2004). A motor capacity of 0.9 MW and 1.2 MW were selected for 6 and 9.29 m, respectively, by choosing the closest next largest market size motor available. The estimated motor size was in the reported range of installed stirrer power 100–1500 kW for large scale bacterial fermentation process (Meyer et al. 2017). In practice, most companies provide the bioreactors at R&D and industrial levels with Ruston turbines due to its wide applicability across systems (liquid–gas, liquid–liquid and liquid–solid) and availability in various configurations with distinct applications (Bustamante et al. 2013). The bioreactor and mixing system design in the current study are in good agreement with previous investigations (Karp et al. 2011; Stitt and Simmons 2011; Meyer et al. 2017; Nienow 2021).

**Fig. 1:**
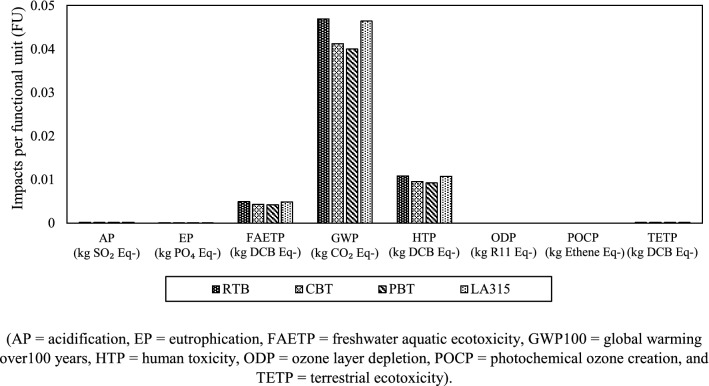
Environmental impacts of different turbines employed in the industrial scale fermenter

**Fig. 2:**
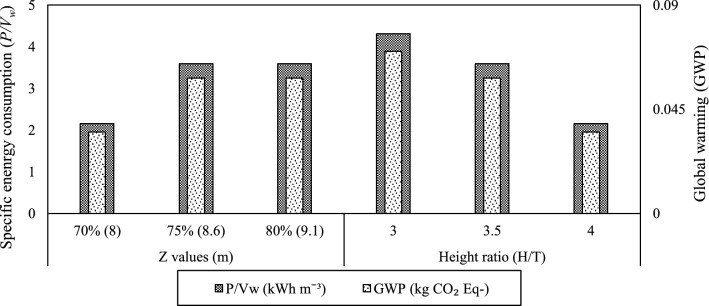
Scenario analysis showing the effect of change in *Z* (working volume height) and *H/T* ratio values on specific energy consumption *(P/V*_w_) and global warming (GWP)

The LCA results indicated that RTB and LA315 turbines cause the greatest environmental impacts especially regarding GWP, whereas CBT and PBT have ~15% lower GWP than RTB and LA315 turbines as shown in Fig. 1. The greater impacts can be attributed to the operational energy consumption for turbines (RTB and LA315), which utilises ~13% more energy than CBT and PBT turbines (Table 4). Similar trends in values were also observed for the turbines across all the environmental impacts considered in the study (Fig. 1). Moreover, a scenario analysis was carried out to assess the impact of changes in fermenter geometric characteristics (*Z* value and *H/T* ratio) on specific energy consumption (*P/V*_w_) and GWP employing RTB turbine operating at 1.33 s^−1^. Figure 2 clearly demonstrates the increase in liquid volume height (*Z*) from 8 to 9.1 m, both *P/V*_w_ and GWP increased up to 8.6 m and above that the values remain constant. For both the liquid volume heights 8.6 and 9.1 m, the probable impellers employed are the same (2 or 3) estimated according to Eq. 4, therefore, suggesting the similar average *P/V*_w_ (3.59 kWh m^−3^) and GWP (0.058 kg CO_2_ equivalent m^−3^) values. However, with the increase in H/T ratio, both *P/V*_w_ and GWP decrease suggesting a reduction in working volume height (*Z*) or tank diameter (T) values. This can be directly linked to the installation of a reduced number of impellers, which leads to lower energy demand and associated emissions.

Efficient resource utilisation and environmental impact reduction are the two factors that dictate the sustainability of a process (Bustamante et al. 2013). To attain a sustainable fermentation process, bio-processors can optimize the inventory (chemicals, enzymes, water etc.,) used in microbial growth medium or electricity consumption during fermenter operation. Life cycle studies indicate that the fermentation stage is a prime contributor to greenhouse gases (GHGs) emissions, either due to resource utilization (e.g., background chemical production emissions) (Bustamante et al. 2013) or electricity consumption (foreground or onsite emissions) (Ruggeri et al. 2011; Kookos et al. 2019). However, from an industrial perspective, energy optimization seems to be the most practical route to emissions reduction as it provides the onsite flexibility to alter the processes as needed at an early design stage. Early stage LCA studies by Ruggeri et al. (2011) and Kookos et al. (2019) were conducted for fermentations processes to assess the critical parameters of bioreactor design that effects the overall emissions of the system. The authors identified that the bioreactor dimensions and mixing system were the major contributors to the global warming impact. The current study results were found to be in line with the above studies as fermenter operations contributed more to GWP than the impacts examined. Optimizing these parameters have significantly reduced the environmental impacts of the system. Hence, performing early stage LCA, offers an opportunity to bioprocess engineers to thoroughly study and identify the key components that effects the environmental impacts, subsequently applying a scaleup strategy to transfer to industrial scale by optimizing the critical parameters for the fermenter design at commercial scale.

## Conclusion

This research demonstrated how to design and scale-up to industrial CSTR fermenter with a capacity of 300 m^3^ based on operational pilot and laboratory scale fermentation testing. The CSTR tank design was accomplished based on geometric similitude, and the impeller design conditions were based on a constant *P/V*_*w*_ strategy. From a range of impeller types (Rushton turbine blade (RTB), Lightnin A310 three blade hydrofoil (LA310), Lightnin A315 four-blade hydrofoil (LA315), concave blade turbine (CBT), pitched blade turbine (PBT) and Marine propeller (MP)) and agitation speeds (0.29–2.5 s^−1^) an RTB type with an impeller speed of 1.33 s^−1^ was found to be the optimal owing to its greater torque generation at slower stirring speed and little changer of cell damage in the microbial biomass. The early stage LCA for the scaled-up fermenter showed a significant link between the number of impellers employed and its associated environmental impacts. Moreover, it identifies the factors (fermenter geometry and dimensions) that can be optimised at an early stage to improve the environmental performance of the system. The proposed fermenter and impeller design will be taken into consideration during the setup of the world’s first-of-a-kind biorefinery with a capacity of 20,000 kg LA yr^−1^ from dairy whey waste.

Despite the key role of scale-up in process development, it appears no specific strategy or approach is commonly recognized which particularly satisfies the practical aspects. This is due to the existence of trade-offs between different parameters (geometric similarity factors and working volume heights; fermenter scales and calculated impeller speeds) that over/underestimates the power requirements than expected. Therefore, this work addresses the limitations prevalent in the application of standard scale-up methodologies and provides a systematic framework for fermenter scale-up followed by selection of suitable turbine type and speed for power estimation at industrial levels. However, further research is required in this area as different fermentation technologies or processes are deemed to have a particular set of parameters that relate to the performance. Understanding and optimising such parameters at each scale of development along with technical experience results in successful scale-up and accurate estimation of energy demand and emissions.

## Supplementary Information

Below is the link to the electronic supplementary material.

Supplementary file1 (DOCX 35 KB)

## Data Availability

The technological data towards the pilot scale fermentation process has not been shared under confidentiality agreement with Glanbia Ireland. Data are however available from the authors upon reasonable request and with permission of Glanbia Ireland. However, the datasets generated through life cycle analysis during the study are available from the corresponding author on reasonable request.
