# Supplementary material for: Upscale fermenter design for lactic acid production from cheese whey permeate focusing on impeller selection and energy optimization
Source: J Food Sci Technol. 2021 Aug 25;59(6):2263–73. doi: 10.1007/s13197-021-05239-6 (PMC9114246; doi:10.1007/s13197-021-05239-6)
Supplement: Supplementary file 1 — Supplementary file1 (DOCX 35 KB) [file 13197_2021_5239_MOESM1_ESM.docx]

**Supplementary Table S1.** Design parameters for lab, pilot and industrial scale fermenters

| **Fermenter dimensions** | **Units** | **Lab scale** | **Pilot scale** | **Industrial scale** | **Basis** | **Reference** |
| --- | --- | --- | --- | --- | --- | --- |
| Total tank volume (*V*_t_) | m^3^ | 0.005 | 0.143 | 300^a^ | Plant site data | AgriChemWhey Project |
| Tank working volume (*V*_w_) | m^3^ | 0.003 | 0.100 | 207 ^a^ | Plant site data |  |
| Tank diameter (*T*) | m | 0.131 | 0.403 | 4.508 ^a^ | Plant site data |  |
| No of bafffles | - | 4.000 | 4.000 | 4.000^a^ | Plant site data |  |
| No. of blades | - | 6.000 | 6.000 | 6.000 ^a^ | Plant site data |  |
| Impeller diameter (*D*) | m | 0.059 | 0.181 | 2.029 | *D/T* = 0.45 | Meyer et al. [9] |
| Tank height (*H*) | m | 0.405 | 1.209 | 13.824 | *H/D* = 3 | Meyer et al. [9] |
| Blade width (*B*_w_) | m | 0.012 | 0.036 | 0.406 | *B*_w_*/D* = 5 | Meyer et al. [9] |
| Blade lenght (*B*_L_) | m | 0.015 | 0.045 | 0.507 | *B*_L_*/D* = 4 | Meyer et al. [9] |
| Baffle distance from the bottom of tank (*T*_b_) | m | 0.029 | 0.091 | 1.014 | *T*_b_*/D* = 0.6 | Meyer et al. [9] |
| Baffle width (*b*) | m | 0.013 | 0.040 | 0.451 | *b* = *D*/10 | Meyer et al. [9] |
| Distance btw baffles and vessel wall (*c*) | m | 0.002 | 0.006 | 0.075 | *c* = *T*/60 | Meyer et al. [9] |
| Distance between impellers (*B*) | m | 0.097 | 0.299 | 3.347 | *B/D* = 1.65 | Fitschen et al. [15] |

^a Information based on industrial experts opinion (proposed for 20,000 ton yr-1 LA production plant)^


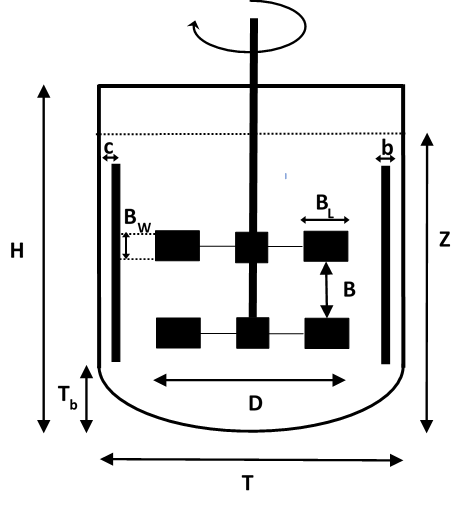


**Supplementary Fig. S1** Proposed industrial scale fermenter for LA production from dairy whey at AgriChemWhey site
